# *N*-acetylcysteine reduces prefrontal reactivity to cocaine cues in individuals with cocaine use disorder

**DOI:** 10.3389/fpsyt.2024.1489194

**Published:** 2025-02-27

**Authors:** Etna J. E. Engeli, Katrin H. Preller, Nathalie M. Rieser, Johanna Klar, Philipp Staempfli, Lea M. Hulka, Matthias Kirschner, Erich Seifritz, Marcus Herdener

**Affiliations:** ^1^ Centre for Addictive Disorders, Department of Adult Psychiatry and Psychotherapy, Psychiatric University Clinic Zurich and University of Zurich, Zurich, Switzerland; ^2^ Pharmaco-Neuroimaging and Cognitive-Emotional Processing, Department of Adult Psychiatry and Psychotherapy, Psychiatric University Clinic Zurich and University of Zurich, Zurich, Switzerland; ^3^ Sports Neuroscience, University of Zurich, Zurich, Switzerland; ^4^ Department of Adult Psychiatry and Psychotherapy, Psychiatric University Clinic Zurich and University of Zurich, Zurich, Switzerland; ^5^ Division of Adult Psychiatry, Department of Psychiatry, Geneva University Hospitals, Geneva, Switzerland; ^6^ Neuroscience Centre Zurich, University of Zurich and Swiss Federal Institute of Technology Zurich, Zurich, Switzerland

**Keywords:** N-acetylcysteine, cocaine use disorder, fMRI, craving, cue reactivity, prefrontal cortex

## Abstract

**Background:**

Individuals with cocaine use disorder experience heightened motivation to pursue rewards tied to cocaine, often triggered by associated cues. Cue reactivity and subsequent craving significantly elevate the risk of substance use, creating a pressing need for treatments that can help alleviate cravings. However, no pharmaceutical therapies for treating cocaine use disorder have been approved. Preclinical findings reveal dysfunctions in the glutamatergic pathway connecting prefrontal regions with the nucleus accumbens, which are correlated with cue-induced substance-seeking behaviour. These alterations, at both molecular and behavioural levels, can be reversed in rodents with *N*-acetylcysteine, a modulator of glutamatergic signalling. In contrast, the therapeutic potential for humans remains uncertain.

**Methods:**

Here, we assessed the impact of a short-term challenge with *N*-acetylcysteine on neural responses to cocaine cues and cue-induced craving in a randomised, placebo-controlled cross-over trial using a fMRI cue reactivity paradigm. In total, 44 fMRI cue reactivity scans of 22 individuals with cocaine use disorder were recorded—once after the administration of 2,400 mg of *N*-acetylcysteine/day for 2 days and once after placebo intake.

**Results:**

In the placebo condition, participants showed increased cue reactivity towards cocaine pictures, accompanied by significantly higher cravings as compared to neutral images. In accordance with recent meta-analyses, cue reactivity was evident in parietal regions such as the posterior cingulate and precuneus, temporal regions like the hippocampus, the bilateral insula, and medial prefrontal regions, namely the inferior, middle, and superior frontal gyrus. Cue-induced activity in the superior frontal gyrus was strongly predicted by the individual duration of cocaine use. While *N*-acetylcysteine showed no impact on subjectively rated cocaine craving, neural cue reactivity in the superior frontal gyrus was significantly decreased under *N*-acetylcysteine compared to placebo.

**Conclusions:**

Our findings show that prefrontal reactivity to cocaine cues can be reduced even by a brief pharmacological challenge with *N*-acetylcysteine. Since neural drug cue reactivity has been shown to be a precursor of relapse behaviour, *N*-acetylcysteine’s therapeutic potential should be further investigated in future studies by extending treatment periods.

**Clinical Trial Registration:**

https://clinicaltrials.gov, identifier NCT02626494.

## Introduction

Vulnerable individuals can lose control over initially recreational substance use and, thus, experience a transition to addiction (1). In individuals with substance use disorder (SUD), repeated substance use has profoundly remodelled the reward system. While it is important for survival to remember cues that predict natural rewards, memories related to substance-induced rewards can become particularly robust and persistently predominant over natural rewards, even after long periods of abstinence (2, 3). The remodelled reward system characterising SUD therefore leads to maladaptive reward-seeking behaviour. This manifests in heightened cue reactivity in individuals with SUD when confronted with substance-associated cues (4–6). Exposure to substance-related cues often leads to craving and potential substance use, even in the face of well-known adverse consequences (5). In addition to the increased pursuit of the psychoactive substance, the interest in natural rewards declines (e.g., food, sex, and social interactions) (7–9). This reward imbalance has been found to be reflected in profound alterations within the frontostriatal network. While the prefrontal cortex (PFC) shows generally reduced basal activity in SUD and when processing naturally rewarding stimuli (4, 7, 8, 10, 11), the responsiveness of the PFC and the nucleus accumbens is heightened when exposed to substance-related stimuli (4, 10, 12, 13), which is linked to increased craving (10, 14). This is in accordance with the notion that the frontostriatal network normally governs adaptive behaviours towards internal and environmental stimuli [for review, see (15)].

As a consequence of chronic cocaine use, adaptations in frontostriatal connectivity lead to hampered prefrontal control over the nucleus accumbens, which translates into difficulty in inhibiting substance-seeking behaviours (16). This might explain why individuals with cocaine use disorder (CUD) cannot effectively regulate cocaine craving and, consequently, choose to use cocaine over other rewarding activities when confronted with cues or stress. The fact that cue-induced craving substantially contributes to the persistence of this disease and leads to chronic progression emphasises the need for a pharmacological intervention with anticraving properties. However, despite the detrimental impact of cocaine use and CUD on individual and public health (17, 18), there is currently no approved pharmacotherapy for cocaine craving or other symptoms of CUD (19). Thus, it is essential to understand the underlying mechanisms of cocaine craving and target the neurobiological underpinnings.

In rodents, dysregulation of glutamatergic signalling between the PFC and the nucleus accumbens appears to be responsible for the maintenance of SUD (20–22). While withdrawal is paralleled by reduced levels of extracellular glutamate in the nucleus accumbens (23), reinstatement of prime- and cue-induced substance-seeking is associated with prefrontal glutamate release (24–30). The consequent spillover of glutamate in the nucleus accumbens is critical for the initiation of relapse (26, 27, 31, 32). These disruptions in glutamate homeostasis were restored, and associated substance-seeking was inhibited by *N*-acetylcysteine, a modulator of glutamate synthesis (N-AC) (21, 22).

In humans diagnosed with CUD (22), similar changes at both the neurometabolic and circuit levels associated with cue exposure and craving experience have been observed (33–35). Individuals with CUD show decreased glutamate concentrations in the ventral and dorsal striatum compared to healthy controls (33, 35), while during cue-induced craving states, glutamate is significantly increased (33). Thus, N-AC has the potential to counterbalance these alterations in glutamatergic signalling underlying craving and, thereby, diminish the vulnerability to relapse in humans with SUD (23, 36–45). Initial pilot studies investigating the therapeutic effect of N-AC on CUD led to promising findings (46–49). While larger placebo-controlled clinical trials in CUD showed beneficial effects on the salience of cocaine cues, cocaine craving, and cocaine use (48–51), the largest clinical trial in CUD failed to demonstrate significant reductions in cocaine use (52). A restoring effect of N-AC on glutamate in the anterior cingulate cortex was reported in CUD by one study (53); conversely, others did not succeed in verifying these observations in individuals who regularly use cocaine (54). Correspondingly, our recent research found no significant impact of N-AC on glutamate in the nucleus accumbens among individuals with CUD (33). Finally, cue reactivity and craving have often been found unaffected, even in studies that reported a beneficial impact on cocaine consumption patterns (51, 55). This is surprising, given that the predictive value of cue reactivity and craving for relapse has been consistently shown across psychoactive substances (56, 57).

Given the promising but inconsistent evidence on N-AC in SUD (33, 46–55), we investigate the impact of a short-term N-AC challenge on cue reactivity in individuals with CUD in a randomised, placebo-controlled, double-blind, cross-over trial. We hypothesised that (i) exposure to cocaine cues, compared to neutral cues, induces changes in the blood oxygen level-dependent (BOLD) signal in the prefrontal-striatal network, which is crucial for cue-induced substance seeking in rodents (22) and other brain regions found to be involved in cue reactivity across psychoactive substances in individuals with SUD (58, 59); and (ii) these changes positively correlate with the severity of CUD (57). Furthermore, we anticipate that N-AC reduces (iii) cue reactivity in the brain and (iv) the subjective experience of craving.

## Materials and methods

### Participants

In total, we recruited 36 participants, of whom 14 had to be excluded due to incomplete or incompliant study participation (for details, see **Supplementary Material**), leaving an effective sample size of *N* = 22. This study sample constitutes a subset of a multimodal study, the findings of which were published previously (33, 34).

General inclusion criterion for all participants was age between 18 and 50 years, and the exclusion criteria included allergy to N-AC, contraindications to magnetic resonance imaging, serious somatic illness, previous head injury, neurological disorders, family history of severe psychiatric disorders according to the Diagnostic and Statistical Manual of Mental Disorders Version IV (DSM-IV) (60), pregnancy, lack of contraception, current participation in another clinical trial, and other current Axis I psychiatric disorders according to DSM-IV, excluding nicotine use disorder and attention-deficit/hyperactivity disorder, as both are highly prevalent in individuals with CUD.

Participants were requested to abstain from alcohol for 48 h and from illicit substances for 72 h prior to both MRI measurement days. Compliance with abstinence was monitored by self-reports and urine samples. Nicotine use was permitted until 1 h before the MRI measurements.

The study was approved by the ethics committee of the Canton of Zurich (No. 2014-0010), and all participants gave written informed consent in accordance with the Declaration of Helsinki prior to study participation. Participants received financial compensation both after completion and after discontinuation of the study.

### Study design

**Figure 1:**
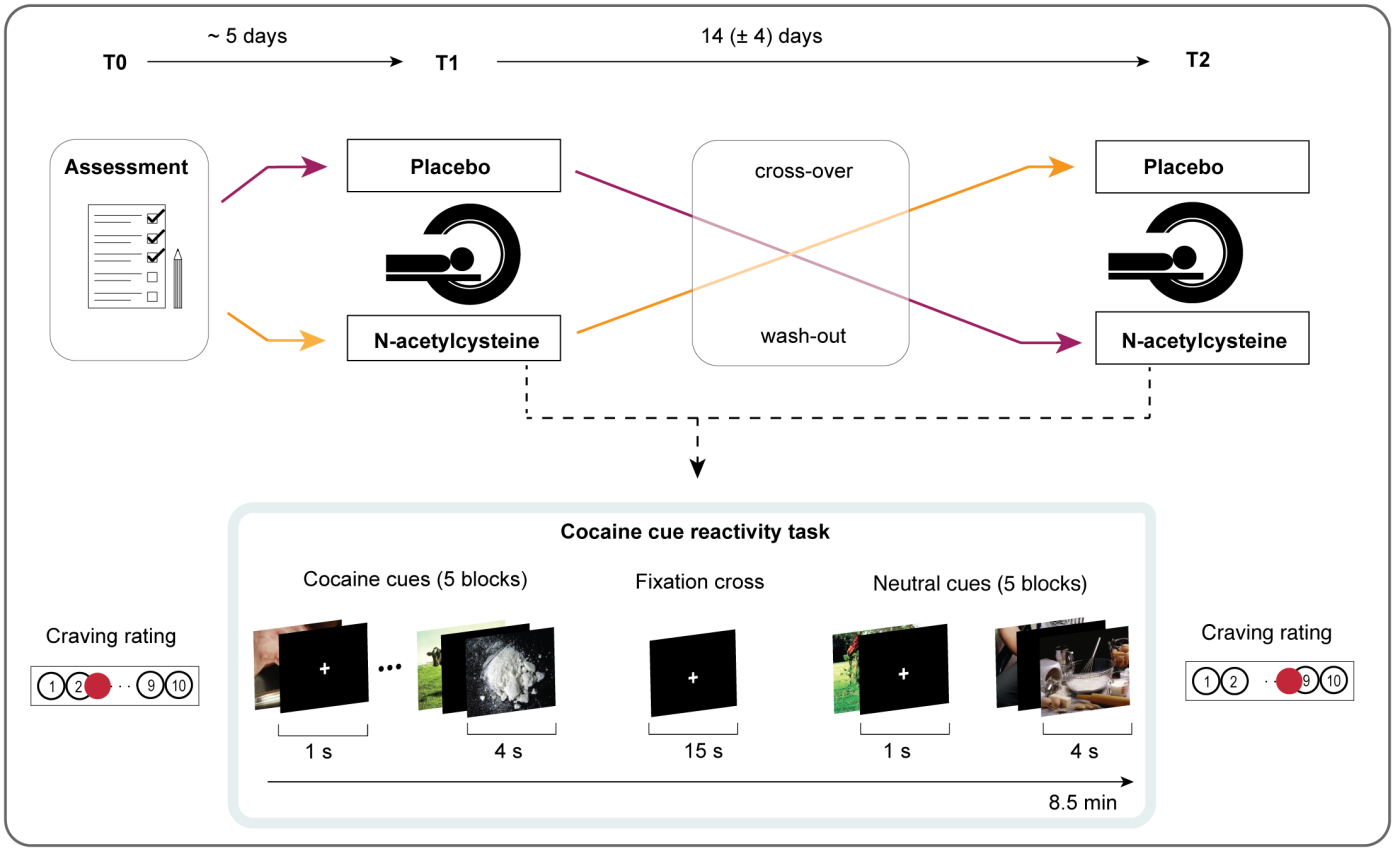
Experimental study design. During the first visit (T0), standardised interviews were conducted, and participants completed questionnaires. Based on double-blinded randomisation, participants either received N-AC or placebo 1 day and 1 h before the first MRI session (T1). After a 14-day washout period, participants were crossed over to receive the other compound for the second MRI session (T2). Both MRI sessions were identical and included a functional magnetic resonance imaging (fMRI) sequence during a cocaine cue reactivity task. Before and after each session, participants reported the intensity of their current cocaine craving on a visual analogue scale (VAS).

This study was designed as a randomised, double-blind, placebo-controlled, cross-over, and counter-balanced investigation (see **Figure 1**). To assess neural cue reactivity under N-AC and compare it to cue reactivity under placebo, two identical measurement sessions were performed and spaced 14 days (± 4) apart to ensure a complete washout of the compound. All subjects received a placebo (1,600 mg mannitol/day) for one of the measurement sessions and N-AC (2,400 mg/day) for the other. Both substances were administered in four identical capsules per day over two consecutive days, 1 day before and on the measurement day. The dose of N-AC was chosen based on previous studies showing that it is well tolerated in this population (21, 46) and may modulate cocaine craving, relapse risk, and glutamate levels (21).

### Questionnaires

At the assessment visit, we collected the following data: demographic data, an interview on consumption patterns of cocaine and other psychoactive substances (61), and a questionnaire assessing general craving over the past weeks by the obsessive-compulsive cocaine scale (OCCUS (62);. All analyses with questionnaires were conducted using R version 4.1.2 within RStudio version 2023.06.0 + 421.

### Cue reactivity fMRI task

We implemented a previously established visual cue reactivity task that allowed us to compare neural responses to cocaine-related pictures with neutral pictures (63, 64). Since substance use behaviours differ strongly between cultures, the task was adapted to ensure authenticity by including locally characteristic cocaine cues. Specifically, seven of the original cocaine-related pictures, which predominantly showed crack cocaine inhalation by people of colour, were replaced with new images showing (i) intranasal cocaine use and the respective paraphernalia (e.g., cocaine powder, banknotes) and (ii) Caucasian protagonists to address the anticipated study population. The visual complexity, attention to detail, luminosity, and composition of both cocaine-related and neutral pictures were matched.

Overall, the picture set consisted of full-colour cocaine-related pictures (*n* = 30) and neutral pictures (*n* = 30), presented in 10 pseudo-randomised blocks, each consisting of seven randomised pictures. The blocks were pseudo-randomised to control for time and expectation effects. To ensure participants’ engagement, they were asked to press a button whenever a target picture of an animal was detected. One target picture was presented in each block at a randomised position (*n* = 10). Every picture was presented for 4 s, with a fixation cross displayed for an average of 1s between pictures (jittered between 750 ms and 1,250 ms). An additional fixation cross appeared for 15 s between blocks. The total task duration was approximately 8.5 min. The task was presented to the participants using MR-compatible goggles (Resonance Technologies, Northridge, CA, USA). Prior to the task, participants were asked to rate their current cocaine craving (precraving) using a visual analogue scale (VAS; 0 = no craving to 10 = strong craving). After the task, participants rated the intensity of the cocaine craving experienced during cue exposure on the VAS (postcraving). To analyse the potential change in cocaine craving due to the cue presentation, we conducted repeated measures analyses of variance (ANOVA) using R version 4.1.2 within RStudio version 2023.06.0 + 421 with *N* = 21 due to one missing value.

### MRI acquisition

All magnetic resonance imaging data were obtained using a Philips Achieva 3-T whole-body scanner (upgraded to the dStream platform) equipped with a 32-channel head coil (Philips Healthcare, Best, The Netherlands). First, high-resolution anatomical images (voxel size: 1 mm × 1 mm × 1 mm) were acquired using a standard T1-weighted 3D turbo field echo sequence. Functional data during the cue reactivity task were acquired using a whole-brain gradient-echo imaging planar (EPI) sequence (repetition time = 2,000 ms, echo time = 35 ms, flip angle = 82°, field of view = 220 mm^2^ × 220 mm^2^, acquisition matrix = 80 × 80, in-plane voxel size reconstructed to 2 mm^2^ × 2 mm^2^, slice thickness = 3 mm, slices = 27, SENSE reduction factor 2.0).

### fMRI: preprocessing and analysis

MRI preprocessing was carried out using SPM8 and analysis with SPM12 (http://fil.ion.ucl.ac.uk/spm), based on MatLab 2023a (The MathWorks, Natick, MA, USA, www.mathworks.com). Following standard procedures, preprocessing included slice-time correction, realignment, spatial normalisation to the standard EPI template of the Montreal Neurological Institute (MNI), and spatial smoothing using a Gaussian kernel of 6 mm full width at half maximum to fulfil the statistical requirements for a general linear model.

Using ART within the CONN toolbox (release 22.a) (65), potential outlier scans were identified as acquisitions with framewise displacement exceeding 0.9 mm or global BOLD signal changes above 5 standard deviations. A reference BOLD image was computed for each subject by averaging all scans excluding outliers. In addition, functional data were denoised using a standard denoising pipeline, which included the regression of potential confounding effects characterised by white matter timeseries (5 CompCor noise components), CSF timeseries (5 CompCor noise components), outlier scans (below 137 factors), motion parameters and their first-order derivatives (12 factors), and linear trends (two factors) within each functional run. This was followed by bandpass frequency filtering of the BOLD timeseries between 0.008 Hz and 0.09 Hz. CompCor noise components within white matter and CSF were estimated by computing the average BOLD signal, as well as the largest principal components orthogonal to the BOLD average, motion parameters, and outlier scans within each subject’s eroded segmentation masks.

In the first-level analysis for each participant, a general linear model was implemented, including the exact onset time for all pictures, which were convolved with a canonical hemodynamic response function. A 128-s high-pass filter was applied to remove low-frequency signal drifts. Regressors were modelled according to an event-related design. For each participant, contrasts *neutral cue > cocaine cue, and cocaine cue > neutral cue* were computed for each pharmacological condition and included in the interaction term *placebo > N-AC* (i.e., [*cocaine cue placebo > neutral cue placebo*] *>* [*cocaine cue N-AC > neutral cue placebo*]).

These individual contrasts were entered into a second-level analysis with a flexible factorial design to test within-group effects of the challenge and the interaction of challenge condition × cue condition using *t*-test, applying Family-Wise Error (FWE) correction for multiple comparisons with a threshold of *p* < 0.05. Effects were first analysed at the whole-brain level and, in a second step, using small-volume correction [SVC; (66)] with *a priori*-defined regions of interest (ROI). The selection of ROI was based on two recent meta-analyses applying different approaches to identify drug cue reactivity in SUD, which included data from over 4,000 or 5,000 participants, respectively (58, 59). All masks for these ROI were created with the WFU PickAtlas (RRID: SCR_007378) according to the AAL3 atlas (Rolls et al., 2020). Detailed descriptions of all ROI, including AAL3 labelling, are presented in the **Supplementary Material**. All brain coordinates are reported in the MNI atlas space.

### Robust regression analysis

A regression model was applied to test whether the severity of CUD predicts the neural reactivity to cocaine cues. During outlier screening, extreme values were identified; however, due to their consistent increase across different variables reflecting CUD severity, they were considered real data rather than measurement errors. Consequently, these data points were included in robust regression, an approach that employs differentiated weighting to mitigate the impact of extreme data points, thereby providing a better fit for the predominant portion of the data. To evaluate different models of robust regression, we compared their residual standard error to assess the standard deviation. We found that for all tested variables, the robust regression based on least-trimmed squares (LTS) showed a lower residual standard error compared to other robust regression estimations, thus providing the best fit to the data.

The variables reflecting CUD severity were general craving (OCCUS), cue-induced craving (VAS), and variables for cocaine use patterns (IPDC).

As measures for cue reactivity, we extracted the first eigenvariates during the condition *cocaine cue placebo* and *neutral cue placebo* in SPM12, without adjustment, as no covariates were included in the general linear model. Two separate robust regression models were applied to test the prediction of the severity of CUD on BOLD response to cocaine cues and to neutral cues. The robust regression model for the difference between *cocaine cue placebo* and *neutral cue placebo* leads to an analogous result (*cocaine cue placebo* > *neutral cue placebo*; see **Supplementary Figure S2** in the **Supplementary Material**).

## Results

### Sample characteristics

As shown in **Table 1**, the final sample consisted of 22 individuals with CUD, including five women. The overall mean age was 30.7 years (SD = 6.1, range: 19.2–45.8). The most common administration route was nasal use (*n* = 19), while three participants predominantly inhaled cocaine. On average, participants used cocaine 3.2 times per week (SD = 1.8, range: 0–7) and consumed a total of 5.7 g/week (SD = 10.9, range: 1–52.5) over the last 6 months. Urine drug screening, with a sensitivity to detect metabolites of psychoactive substances between days and weeks following the last use, identified cocaine metabolites in probes of eight participants before the placebo measurement and in the probes of 11 participants before the N-AC measurement. However, self-reports indicated that only three participants had used cocaine within 3 days prior to the placebo measurement and two before the N-AC measurement. Overall, other substances detectable with the applied urine drug screening (amphetamine, benzodiazepines, cannabis, and opiates) yielded positive results in six participants before the placebo measurement and in seven participants before the N-AC measurement. According to self-reports, two participants used another psychoactive substance (including amphetamine, MDMA, psychedelics, ketamine, GHB, opiates, and opioids) within 3 days before the placebo measurement and four participants before the N-AC measurement. All participants reported no alcohol use in the 2 days leading up to the placebo measurement, while one participant indicated having drunk alcohol before the N-AC measurement. Further information regarding the use of other psychoactive substances is summarised in **Table 1**.

**Table 1:** Sample characteristics and patterns of substance use in individuals with CUD.

	Individuals with CUD (*N* = 22)
	Mean (SD)
Male/female	16/6
Age in years	30.72 (6.07)
OCCUS	20.65 (7.89)
Cocaine	
Grams/week	5.68 (10.92)
Frequency/week	3.16 (1.81)
Duration of use (years)	6.53 ± 3.96
Cumulative dose (grams/lifetime)	1,989.92 (2,528.32)
Alcohol	
Grams/week	310.65 (581.97)
Nicotine	
Cigarettes/week	97.91 (72.97)
Cannabis	
Grams/week	1.96 (5.96)
Amphetamine	
Grams/week	0.17 (0.58)
MDMA	
Milligrams/week	22.80 (44.22)
Opioid	
Grams/week	0.02 (0.05)
GHB	
Millilitres/week	0.01 (0.03)
Hallucinogens	
Trips/week	0.17 (0.64)
Ketamine	
Grams/week	0.03 ± 0.07

Means and standard deviations (SD) of sample characteristics assessed using the obsessive–compulsive cocaine use (OCCUS) questionnaire, which ranges from 0 to 56, and the interview on psychotropic drug consumption.

*MDMA*, 3,4-methylenedioxymethamphetamine; *GHB*, gamma-hydroxybutyric acid.

### Cue-induced craving

After the cue reactivity task, craving levels were significantly increased compared to before the task, as indicated by self-reports in the placebo condition on the VAS (precraving: M = 3.41, SE = 0.57; postcraving: M = 4.27, SE = 0.65; main effect cue: F = 8.28, *p* = 0.009, *N* = 21). Contrary to our hypothesis, N-AC did not lead to a significant reduction in subjective craving levels (precraving: M = 3.91, SE = 0.65; postcraving: M = 4.92, SE = 0.62; main effect of challenge: F = 3.94, *p* =0.15; cue-by-challenge interaction effect: F = 0.0004, *p* = 0.98, *N* = 21).

### Cue reactivity in the brain

**Figure 2:**
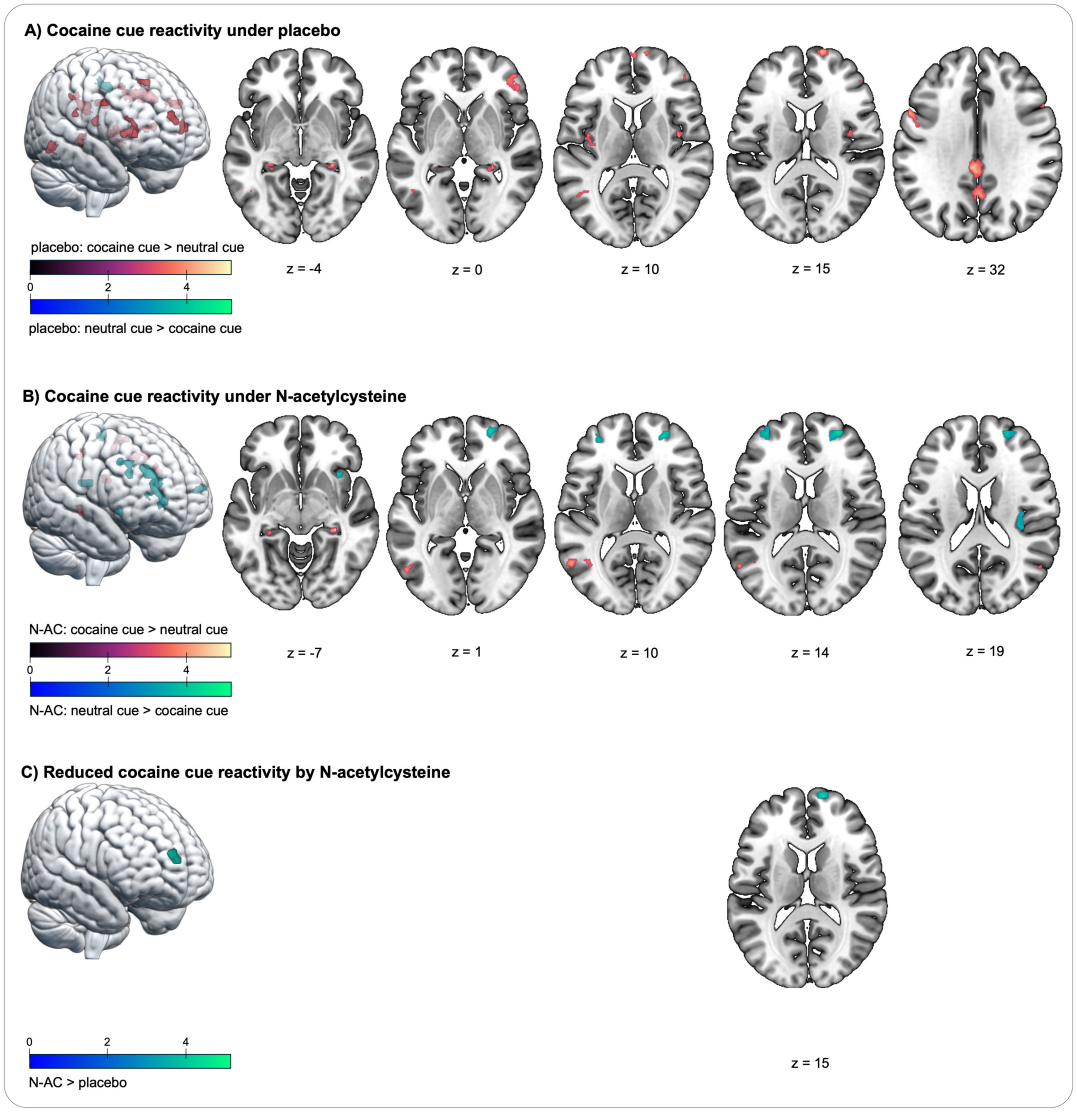
Changes in blood oxygenation level-dependent (BOLD) signal during the cocaine cue reactivity task in individuals with cocaine use disorder. **(A)** Cocaine cue reactivity increases the BOLD signal under placebo in four prefrontal regions, spanning the superior frontal gyrus, medial frontal gyrus, and inferior frontal gyrus; in the bilateral insula and hippocampus; in the postcentral gyrus; in the posterior cingulate cortex; in the supramarginal gyrus; in the precuneus, and in additional temporal and parietal regions (contrast cocaine cue placebo > neutral cue placebo). **(B)** Cocaine cue reactivity increases the BOLD signal under placebo in regions similar to those in the placebo condition, including the bilateral hippocampus, precuneus, and temporal and parietal regions (contrast cocaine cue N-AC > neutral cue N-AC). In contrast, there is reduced BOLD signal in three prefrontal regions and in the insula. **(C)** A cue-by-challenge interaction effect shows significantly increased BOLD signal in a medial prefrontal region during placebo compared to N-AC, as indicated by the contrast (cocaine cue placebo > neutral cue placebo) > (cocaine cue N-AC > neutral cue N-AC). The colour spectra represent the *t*-scores.

In contrast, *cocaine cue placebo > neutral cue placebo* revealed significantly increased cue reactivity, predominantly in the prefrontal cortex, bilaterally in the precentral gyrus/inferior frontal gyrus (IFG), in the right IFC/middle frontal gyrus, and in three clusters of the superior frontal gyrus (SFG), including lateral and dorsal activation, as well as a cluster in the medial frontal pole. Furthermore, significantly elevated cue reactivity was observed in the bilateral posterior insula, bilateral hippocampus, precuneus, posterior and medial cingulate cortex, postcentral gyrus, and two clusters in the middle temporal gyrus. For an overview of regions with significantly increased BOLD signals, along with their respective coordinates and statistical values, see **Table 2** and **Figure 2A**.

**Table 2:** Brain regions exhibiting increased cocaine cue reactivity under placebo conditions.

Coordinates (mm)			Brain region	Side	Cluster size	Statistics			SVC (*p*-value)
*x*	*y*	*z*				*t*	*z*	FWE (*p*-value)	
Cocaine cue placebo > neutral cue placebo									
12	68	12	Superior frontal gyrus	R	54	4.61	4.34	0.067	**0.01**
− 2	68	6	Superior frontal gyrus	L/R	33	4.23	4.02	0.304	**0.046**
22	36	50	Superior frontal gyrus	R	35	4.32	4.09	0.263	**0.022**
42	40	2	Inferior frontal gyrus	R	126	3.95	3.77	**0.001**	
			Middle frontal gyrus						
56	16	28	Inferior frontal gyrus	R	20	3.84	3.68	0.703	**0.019**
			Precentral gyrus						
− 56	10	34	Inferior frontal gyrus	L	49	4.37	4.14	0.095	**0.006**
			Precentral gyrus						
− 38	− 8	12	Insula posterior	L	34	4.12	3.92	0.283	**0.021**
42	− 8	14	Insula posterior	R	27	4.74	4.45	0.461	**0.002**
− 52	− 28	42	Postcentral gyrus	L	287	4.76	4.61	**0.000**	
			Supramarginal gyrus						
− 4	− 34	32	Posterior cingulate gyrus	L/R	77	4.9	4.58	**0.014**	
			Middle cingulate gyrus						
30	− 34	− 4	Hippocampus	R	38	4.56	4.3	0.212	**0.005**
− 26	− 34	− 6	Hippocampus	L	12	4.13	3.93	0.947	**0.042**
58	− 44	− 8	Middle temporal gyrus	R	53	4.4	4.16	0.072	**0.032**
			Inferior temporal gyrus						
2	− 54	30	Precuneus	L/R	134	5.08	4.34	**0.000**	
			Posterior cingulate gyrus						
− 50	− 66	6	Middle temporal gyrus	L	124	4.76	4.47	**0.001**	
			Occipital lobe						
Neutral cue placebo > cocaine cue placebo									
− 12	− 38	46	Precuneus		92	4.56	4.29	**0.005**	

For both whole-brain analysis and small volume correction (SVC), the significance threshold was set at *p* < 0.05 at the cluster level, with family-wise error (FWE) correction for multiple comparisons, an initial voxel-level threshold of *p* (uncorrected) < 0.001, and an extent threshold of *k* = 10 voxels. *N* = 22. *L*, left; *R*, right. Significant p-values are indicated in bold.

Notably, no significantly enhanced neural cue reactivity was observed in the contrast *cocaine cue placebo > neutral cue placebo* in the following regions, as assessed with SVC: amygdala, anterior cingulate cortex, pallidum, putamen, nucleus accumbens, substantia nigra, thalamus, and ventral tegmental area.

### Cue reactivity in the brain under *N*-acetylcysteine

The contrast *cocaine cue N-AC > neutral cue N-AC* indicates how neural cue reactivity manifests under the influence of N-AC. In the N-AC condition, significantly increased neural cue reactivity was observed in a fewer activity patterns compared to the placebo condition, including the postcentral gyrus, supramarginal gyrus, bilateral hippocampus, precuneus, and two clusters in the middle temporal gyrus. In contrast, significantly reduced BOLD signals were observed in the medial PFC, including the bilateral SFG/MFG, another lateral MFG cluster, and in both the anterior and posterior insula (see **Table 3**; **Figure 2B**).

**Table 3:** Brain regions with increased cocaine cue reactivity following *N*-acetylcysteine.

Coordinates (mm)			Brain region	Side	Cluster size	Statistics			SVC (*p*-value)
*x*	*y*	*z*				*t*	*z*	FEW (*p*-value)	
Cocaine cue N-AC > neutral cue N-AC									
− 54	− 16	26	Postcentral gyrus	L	96	5.41	4.99	**0.004**	
			Supramarginal gyrus						
32	− 32	− 6	Hippocampus	R	34	5.55	5.11	0.283	**0.005**
− 26	− 34	− 4	Hippocampus	L	13	3.92	3.75	0.926	**0.033**
− 50	− 38	44	Supramarginal gyrus	L	58	4.37	4.13	0.050	**0.029**
12	− 52	44	Precuneus	R/L	36	4.05	3.86	0.245	**0.019**
− 58	− 62	12	Middle temporal gyrus	L	29	4.14	3.94	0.402	**0.025**
−54	− 66	0	Middle temporal gyrus	L	32	3.98	3.8	0.326	**0.023**
Neutral cue N-AC > cocaine cue N-AC									
24	58	− 4	Superior frontal gyrus	R	289	5.32	4.92	**0.000**	
			Middle frontal gyrus						
− 36	52	14	Superior frontal gyrus	L	48	3.95	3.77	**0.102**	**0.005**
			Middle frontal gyrus						
38	32	24	Middle frontal gyrus	R	79	4.66	4.39	0.012	
34	18	− 6	Insula anterior	R	27	4.21	4	0.461	**0.015**
34	− 18	18	Insula posterior	R	56	4.69	4.4	0.058	**0.006**
Placebo > N-AC									
14	64	14	Superior frontal gyrus	R	69	4.57	4.3	**0.024**	

For both whole-brain analysis and small volume correction (SVC), the significance threshold was set at *p* < 0.05 cluster level, with family-wise error (FWE) correction for multiple comparisons, an initial voxel-level threshold of *p* (uncorrected) < 0.001, and an extent threshold of *k* = 10 voxels. *N* = 22. *N-AC*, *N*-acetylcysteine; *L*, left; r: right. Significant p-values are indicated in bold.

The comparison between the contrast (*cocaine cue N-AC > neutral cue N-AC*) *>* (*cocaine cue placebo > neutral cue placebo*) revealed a cue-by-challenge interaction in a cluster within the medial PFC, with neural cue reactivity being significantly reduced by N-AC (see **Table 3**; **Figure 2C**). In other *a priori*-defined ROI, no significant interaction effects were observed.

### The link between CUD severity and cue reactivity

The LTS regression model was not significant for the subjective experience of general and cue-induced craving (OCCUS; VAS) or neural cue reactivity in the medial PFC (SFG; 14 64 14) under placebo (first eigenvariates extracted from the condition *cocaine cue placebo* [*k* = 54]).

**Figure 3:**
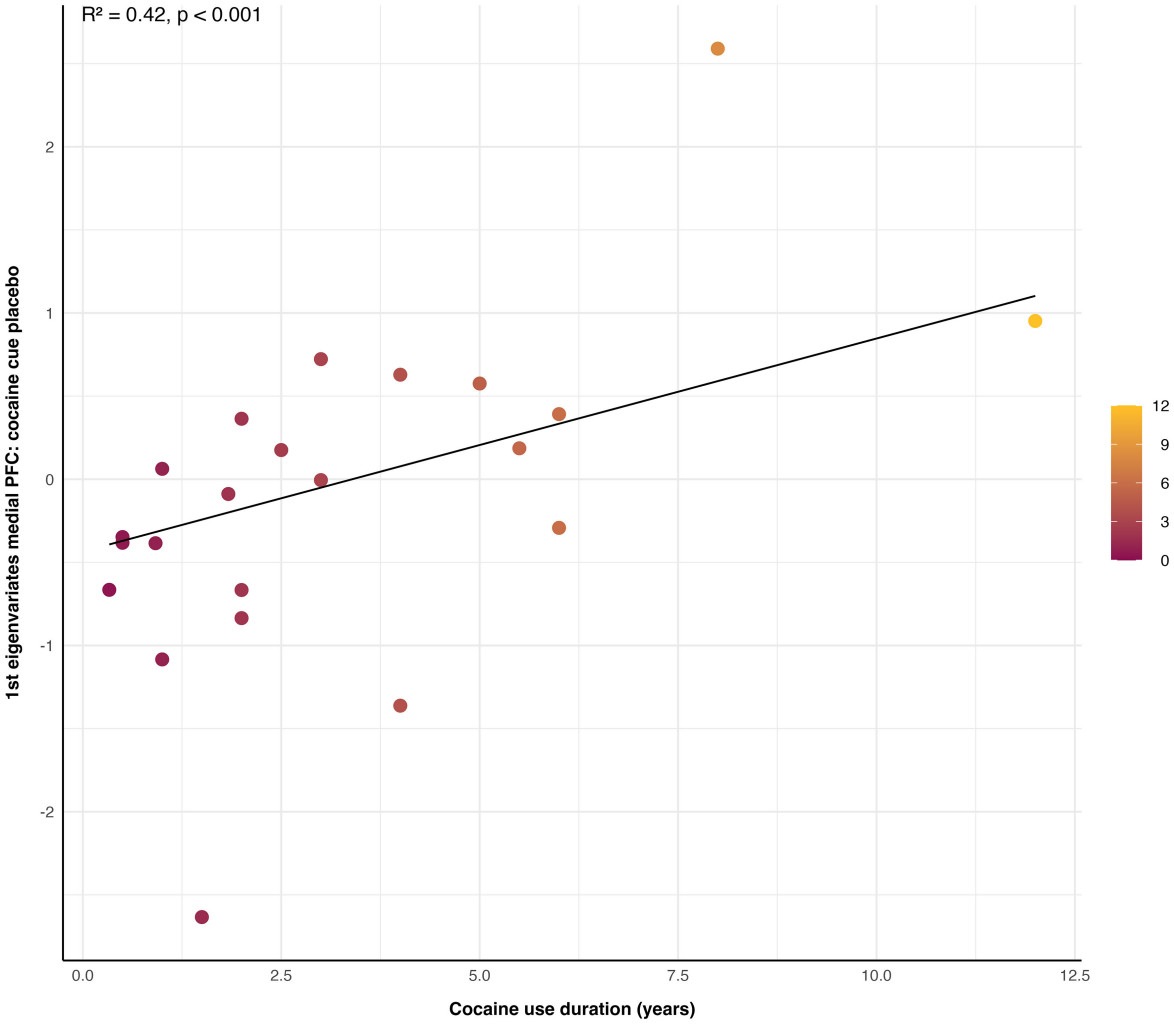
Link between cocaine cue reactivity in the medial prefrontal cortex (PFC) and duration of cocaine use. The duration of cocaine use in years significantly predicted neural cue reactivity to cocaine stimuli within the medial PFC under placebo, as measured by first eigenvariates extracted from the condition *cocaine cue placebo* (*R* ^2^ = 0.42, *p* < 0.001, *N* = 22). The colour shading on the graph represents the duration of cocaine use in years.

In contrast, the cocaine consumption pattern, reflected by the duration of cocaine use (IPDC; self-reports), significantly predicted reactivity to cocaine cues within the medial PFC under placebo, showing a strong prediction of reactivity to cocaine cues (Intercept = − 0.44, *x* = 0.13; *R* ^2^ = 0.42, *p* < 0.001, *N* = 22, **Figure 3**). No significant relationship was observed between the duration of cocaine use and reactivity to neutral cues (for results, see **Supplementary Figure S1**).

## Discussion

This study confirmed increased neural cue reactivity in response to cocaine stimuli among individuals with CUD in most of the neural networks established by extensive meta-analyses (58, 59), with a strong focus on the PFC (67). Most importantly, the cue-induced increase in activity in one of the prefrontal clusters, which was strongly predicted by the individual duration of cocaine use, was significantly reduced by a short-term challenge of N-AC (2,400 mg of N-AC on two consecutive days). This provides the first evidence demonstrating the modulatory impact of N-AC on neural cue reactivity, a strong risk factor for relapse in CUD (56, 68), while there was no effect on the subjective experience of craving.

These findings are in line with consistent preclinical data suggesting that dysfunctional glutamatergic signalling between the PFC and the nucleus accumbens underlies cue-induced urges to seek addictive substances (for overview see (22, 69). N-AC prevents relapse in animals by restoring substance-induced neuroplastic changes in the glutamate system within the PFC-nucleus accumbens pathway (22). The involvement of the PFC in reward processing of both natural and substance-related rewards has become evident through neuroimaging studies emerging over the last two decades (70, 71). The PFC attributes value to stimuli and actions, aiding in the selection of adaptive actions. It is also engaged when the devaluation of stimuli–outcome association is required, allowing for appropriate action switching when circumstances change. The goal-directed behaviour enables efficient functioning in our environment but is often severely disrupted in psychiatric disorders (71). Within the context of SUD, the inability of individuals to devalue previous cue–action coupling may play a significant role in their challenge to modify behaviours appropriately when required to prevent adverse outcomes. As a result, individuals continue engaging in addictive behaviours despite being aware of the high likelihood of experiencing long-term negative physical and psychological effects. This notion is consistent with prevailing evidence that natural rewards elicit decreased prefrontal cortex activity, whereas substance-related cues trigger an amplified response in the PFC, contributing to compulsive urges to use a substance in individuals with SUD despite the adverse consequences.

Prefrontal reactivity observed here is consistent with recent meta-analyses of neuroimaging studies investigating cue reactivity across different SUDs, which showed relatively robust heightened activity in response to substance-related cues in the PFC, including clusters in the medial and dorsolateral PFC, anterior cingulate, and orbitofrontal cortex (58, 59). A transdiagnostic study aimed at identifying the most effective target for neuromodulation of craving found the largest percentage of cue-induced activation in the PFC, specifically in the frontal pole, for cocaine alone and across substances (72). This region overlaps with the medial PFC cluster, which shows increased neural cue reactivity in the placebo condition, is predicted by the duration of cocaine use, and was significantly reduced by N-AC. Furthermore, it has been shown to be an effective target for transcranial magnetic stimulation, with reduced functional connectivity during cue exposure in individuals with CUD and alcohol use disorder (73).

Contrary to previous studies and to our *a priori* hypothesis, we found no significant neural cue reactivity to cocaine stimuli in other key areas of the reward system associated with SUD, despite consistent reports of neural cue reactivity in the striatum, ventral tegmental area, thalamus, and other regions (for an overview, see (58, 59). For example, a study investigating the effect of modafinil on neural cue reactivity with the same task as in the present study found heightened activity in the medial PFC and in the ventral tegmental area under placebo (63). Similar to our observation, prefrontal reactivity was reduced after pharmacological modulation (63). However, a comparison of three studies that aggregated cue reactivity data from individuals with SUD reveals substantial differences. One meta-analysis identified a broad range of brain regions activated by drug cues (59), while others reported drug cue reactivity limited to a few regions, notably excluding the nucleus accumbens (58, 72). These significant discrepancies may stem from variations in methodological approaches and differences in the populations studied. An early review highlighted inconsistencies across studies, suggesting that participant characteristics, such as treatment status, may contribute to divergent findings in cue reactivity research (74). Conversely, given the substantial variability in the results, the identification of a common substrate for cue reactivity in the medial and dorsolateral PFC across different psychoactive substances—which aligns with our results—is particularly noteworthy (58, 59, 72).

Examining the pharmacological conditions separately, exposure to cocaine cues activated certain brain regions similarly, but the overall brain response patterns varied. Prefrontal cue reactivity, which was prominent in the placebo condition (cocaine cues placebo > neutral cues placebo), was absent with N-AC. Instead, N-AC significantly reduced reactivity to cocaine cues in three PFC clusters (cocaine cues N-AC > neutral cues N-AC). Additionally, while the placebo condition heightened reactivity in the bilateral posterior insula (cocaine cues placebo > neutral cues placebo), N-AC led to significantly decreased reactivity in both the posterior and anterior insula (cocaine cues N-AC > neutral cues N-AC). The insula, a key region in introspection and self-awareness, is notably involved in SUD, as evidenced by findings that nicotine use disorder ceased immediately in individuals with insula damage (75) and that decision-making in SUD is characterised by reduced insula engagement, which predicted relapse (76). Notably, these differences in cue reactivity were observed only when analysing the pharmacological contrasts separately, not in a direct statistical comparison of the two pharmacological conditions. Larger sample sizes may be necessary to clarify subtle differences between the groups.

In contrast to other studies demonstrating a significant reduction of cocaine use and cocaine craving after N-AC administration (46–51), we observed only a change in neural cue reactivity but no impact of N-AC on the subjective experience of craving for cocaine. Notably, most of the recent clinical trials report a distinct impact of N-AC on some but not all outcome measures; i.e., in a clinical trial with 24 individuals with CUD, N-AC over 25 days had a beneficial effect on cocaine use and CUD severity but no effect on cue reactivity and cocaine craving (51, 54, 55). In a large sample of over 150 individuals with methamphetamine use disorder, N-AC showed a therapeutic effect, albeit to the same extent as a placebo (77), while N-AC intake over 28 days had a reducing effect on some but not all measures of alcohol use in two different studies with individuals with alcohol use disorder (78, 79). Consequently, the effect of N-AC does not consistently manifest across all clinically relevant outcomes measured. Given that some of the studies that yielded therapeutic effects adopted longer N-AC interventions over several weeks (51, 78, 79), an extended duration of N-AC treatment may be necessary to effectively promote behavioural change in individuals with CUD.

However, a randomised clinical trial with a large sample size administering N-AC for 8 weeks reported no impact of N-AC on overall cocaine use measured by urine analysis (52). Yet, in individuals who were already abstinent before N-AC treatment, the time to relapse was significantly prolonged (52). This finding is consistent with preclinical data showing facilitation of self- but not experimenter-imposed abstinence in rodents (36) and extending time to relapse (45), suggesting that N-AC is primarily effective in maintaining already achieved abstinence, while it might be ineffective in individuals who are currently still using cocaine (80).

Accordingly, we acknowledge the following limitations in this study: a restricted sample size that limits the investigation of subgroups such as responders vs. nonresponders or gender groups, a short period of N-AC administration lasting 2 days, and the lack of assessment of long-term behavioural outcomes. To better understand the therapeutic potential of N-AC, future trials with larger samples to cover the population’s inherent heterogeneity, applying long-term interventions and assessing longitudinal outcomes, are needed. This will allow for the examination of distinct subgroups and the evaluation of clinically relevant treatment effects over time. In this context, the impact of a pharmacological challenge with N-AC on cue reactivity may have the potential to serve as a biomarker for more stratified interventions.

Nonetheless, these findings demonstrate that N-AC, a glutamatergic agent, effectively modulates the neural response to cocaine cues in the medial PFC of individuals with CUD. Thus, N-AC can reduce neural cue reactivity, which typically occurs early in the process of substance use initiation, by affecting the medial PFC, a brain region involved in cue evaluation and action selection. Recent meta-analyses indicate that neural cue reactivity and subsequent subjective experience of craving significantly increase the likelihood of substance use in individuals with SUD, highlighting that managing neural cue reactivity can be crucial in supporting patients’ recovery (56, 68, 81).

Considering the inconsistent findings from the present and previous research, it may be essential to explore the effectiveness of integrating N-AC into a personalised therapeutic approach. For example, transcranial magnetic stimulation of the medial or dorsolateral PFC and neurofeedback training to reduce substance cue reactivity or increase sensitivity to natural rewards have shown promising results in SUD (82, 83). Therefore, a combined pharmacotherapeutic approach involving N-AC and neuromodulation could synergistically enhance adaptive reward processing and experiences, effectively addressing the reward imbalance in SUD treatment.

## Data Availability

The raw data supporting the conclusions of this article will be made available by the authors upon reasonable request.
