# Supplementary material for: N-acetylcysteine reduces prefrontal reactivity to cocaine cues in individuals with cocaine use disorder
Source: Front Psychiatry. 2025 Feb 27;15:1489194. doi: 10.3389/fpsyt.2024.1489194 (PMC11903417; doi:10.3389/fpsyt.2024.1489194)
Supplement: Supplementary file 1 [file DataSheet1.pdf]

Supplementary Material

N-acetylcysteine reduces prefrontal reactivity to cocaine cues in individuals with cocaine use disorder

Etna J. E. Engeli[1], Katrin H. Preller[2], Nathalie M. Rieser[1,2], Johanna Klar[1,3], Philipp Staempfli[4], Lea M. Hulka[1], Matthias Kirschner[1,5], Erich Seifritz[4,6], Marcus Herdener[1]

[1] Centre for Addictive Disorders, Department of Adult Psychiatry and Psychotherapy, Psychiatric University Clinic Zurich and University of Zurich, Zurich, Switzerland.

[2] Pharmaco-Neuroimaging and Cognitive-Emotional Processing, Department of Adult Psychiatry and Psychotherapy, Psychiatric University Clinic Zurich and University of Zurich, Zurich, Switzerland.

[3] University Hospital of Child and Adolescent Psychiatry and Psychotherapy, University of Bern, Bern, Switzerland.

[4] Department of Adult Psychiatry and Psychotherapy, Psychiatric University Clinic Zurich and University of Zurich, Zurich, Switzerland.

[5] Division of Adult Psychiatry, Department of Psychiatry, Geneva University Hospitals, Geneva, Switzerland.

[6] Neuroscience Centre Zurich, University of Zurich and Swiss Federal Institute of Technology Zurich, Zurich, Switzerland.

Materials and Methods

Participants

Participants were recruited through online and in-house advertisement. A total of 36 individuals with cocaine use disorder (CUD) were randomised into the study (03/2015 – 04/2016). Since this sample was part of a multimodal study with a focus on proton magnetic resonance imaging (^{1}H-MRS), the definition of the sample size was made using a power analysis based on a previous ^{1}H-MRS study of glutamate in cocaine users in two prefrontal regions (1).

We anticipated a dropout rate of 30%, while the actual data loss for the present analyses was 39%. Fourteen data sets had to be excluded from the analyses based on the following reasons: Two individuals with CUD dropped out of study participation due to claustrophobia in the MRI scanner. One individual with CUD terminated the study participation because of demotivation, one individual with CUD reporting scheduling difficulties, and one individual with CUD due to acute illness. Two participants did not show up to the second session for unknown reasons. Two individuals with CUD started new pharmacotherapies (methylphenidate) during study participation. Hair sample of one individual with CUD revealed that mean cocaine concentration in hair during the last six months was below the threshold of reliable detection < 500 mg/mg and was therefore excluded from the analysis. Hair analysis and results are described in a previous publication (2). Two individuals with CUD were asleep during the measurement and were therefore omitted. Two individuals with CUD could not be measured at the second MRI session due to technical problems with the MRI scanner.

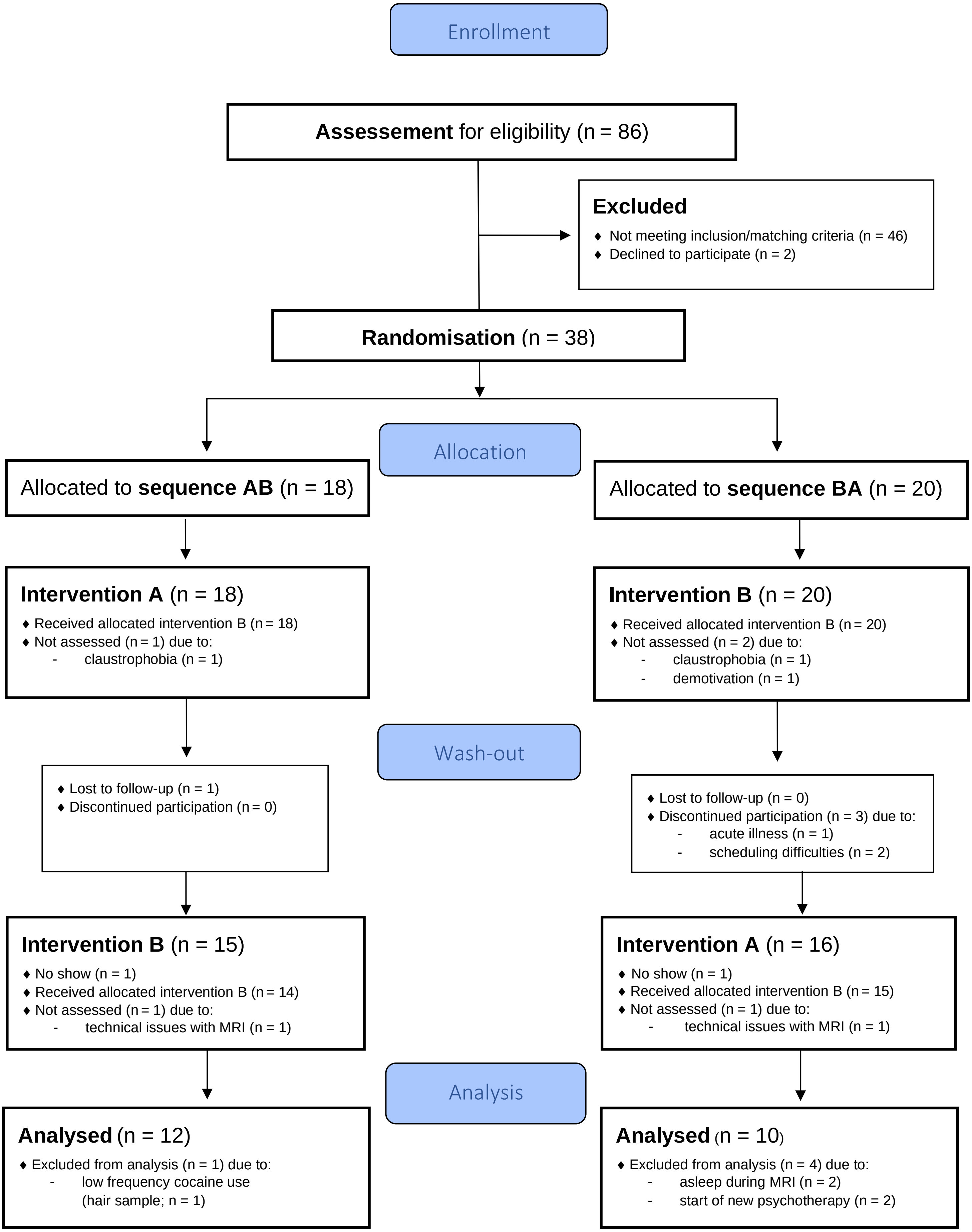

A = placebo, B = N-acetylcysteine

Analysis

Table S1 | Regions of interest with corresponding labels in the AAL3 atlas.

Regions of interest (ROI)	AAL3 labelling
Amygdala	Amygdala_L 45<br>Amygdala_R 46
Anterior cingulate cortex	ACC_sub_L 151 + ACC_pre_L 153 + ACC_sup_L 155<br>ACC_sub_R 152 + ACC_pre_R 154 + ACC_sup_R 156
Frontal middle gyrus	Frontal_Mid_2_L 5<br>Frontal_Mid_2_R 6
Frontal medial gyrus	Frontal_Med_Orb_L_21<br>Frontal_Med_Orb_R_22
Frontal inferior gyrus	Frontal_Inf_Oper_L 7<br>Frontal_Inf_Oper_R 8
Frontal superior gyrus	Frontal_Sup_Medial_L 19<br>Frontal_Sup_Medial_R 20
Hippocampus	Hippocampus_L 41<br>Hippocampus_R 42
Insula	Insula_L 33<br>Insula_R 34
Nucleus accumbens	N_Acc_L 157<br>N_Acc_R 158
Orbitofrontal cortex	OFCmed_L 25 + OFCant_L 27 + OFCpost_L 29 + OFClat_L 31<br>OFCmed_R 26 + OFCant_R 28 + OFCpost_R 30 + OFClat_R 32
Pallidum	Pallidum_L_79<br>Pallidum_R_80
Parietal superior gyrus	Parietal_Sup_L_63<br>Parietal_Sup_R_64
Precentral gyrus	Precentral_L_1<br>Precentral_R_2
Precuneus	Precuneus_L_71<br>Precuneus_R_72
Postcentral gyrus	Postcentral_L_61<br>Postcentral_R_62
Posterior cingulate cortex	Cingulate_Post_L 39<br>Cingulate_Post_R 40
Putamen	Putamen_L 77<br>Putamen_R 78
Supramarginal gyrus	SupraMarginal_L_67<br>SupraMarginal_R_68
Substantia nigra	SN_pc_L 161 + SN_pr_L 163<br>SN_pc_R 162 + SN_pr_R 164
Temporal middle gyrus	Temporal_Mid_L_89<br>Temporal_Mid_L_90
Thalamus (anterior-medial part)	Thal_AV_L 121 + Thal_VA_L 125 + Thal_MDm_L 135<br>Thal_AV_R 122 + Thal_VA_R 126 + Thal_MDm_R 136
Ventral tegmental area	VTA_L 159<br>VTA_R 160

The regions of interest (ROI) listed on the left were a priori defined based on two large meta-analyses. On the right, the corresponding label of the AAL3 atlas (Rolls et al., 2020) implemented in the WFU Pickatlas (RRID:SCR_007378) is presented.

Results

In contrast to the prediction of cocaine cue reactivity, there was not significant association between the duration of cocaine use and blood oxygen level-dependent (BOLD) response to neutral cues in the medial PFC (SFG; 14 64 14) under placebo (first eigenvariates extracted from the condition *neutral cue placebo* (k = 54).) This was tested with a robust regression model based on based on least-trimmed squares (LTS): R2 = 0.003, F = 0.062, p = 0.81, N = 22.

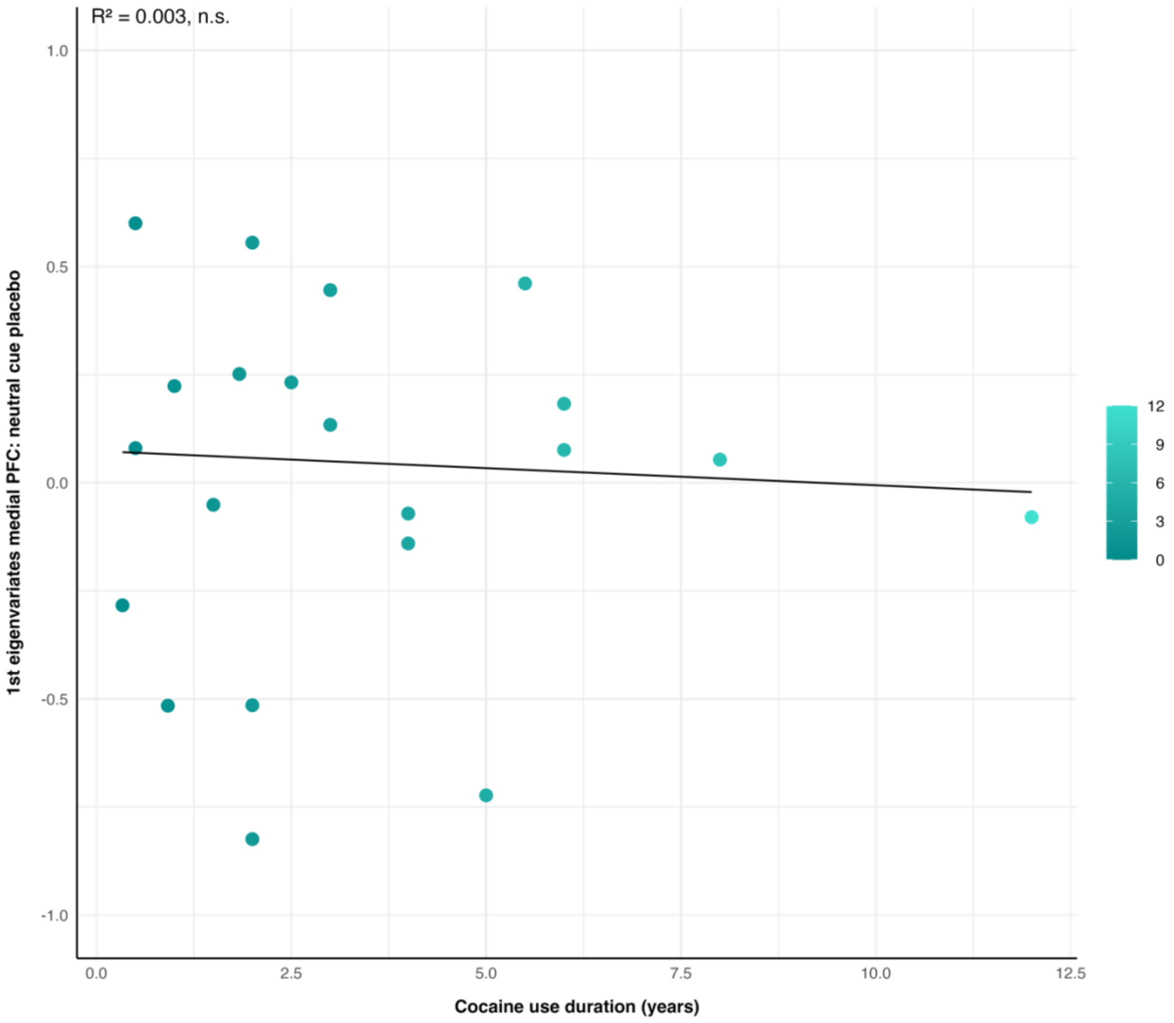

Figure S1. Prediction of neutral cue reactivity in the medial prefrontal cortex (PCF) by duration of cocaine use. Robust regression revealed no significant link between cocaine use duration and brain response in the medial PFC when exposed to neutral cues. The color shading on the graph represents the duration of cocaine use in years.

Although we interpreted all data as real data and applied robust regression to account for potential outliers, we further tested the relationship between duration of cocaine use and neural cue reactivity by excluding one extreme value (duration of cocaine use = 12 years, being 1.5

times the interquartile range greater than the third quartile). After exclusion of the extreme value, the robust regression model based on LTS estimates showed a significant prediction of medium strength for neural cue reactivity by cocaine use duration in years (R2 = 0.30, F = 6.96, p = 0.018, N = 21; Figure S2).

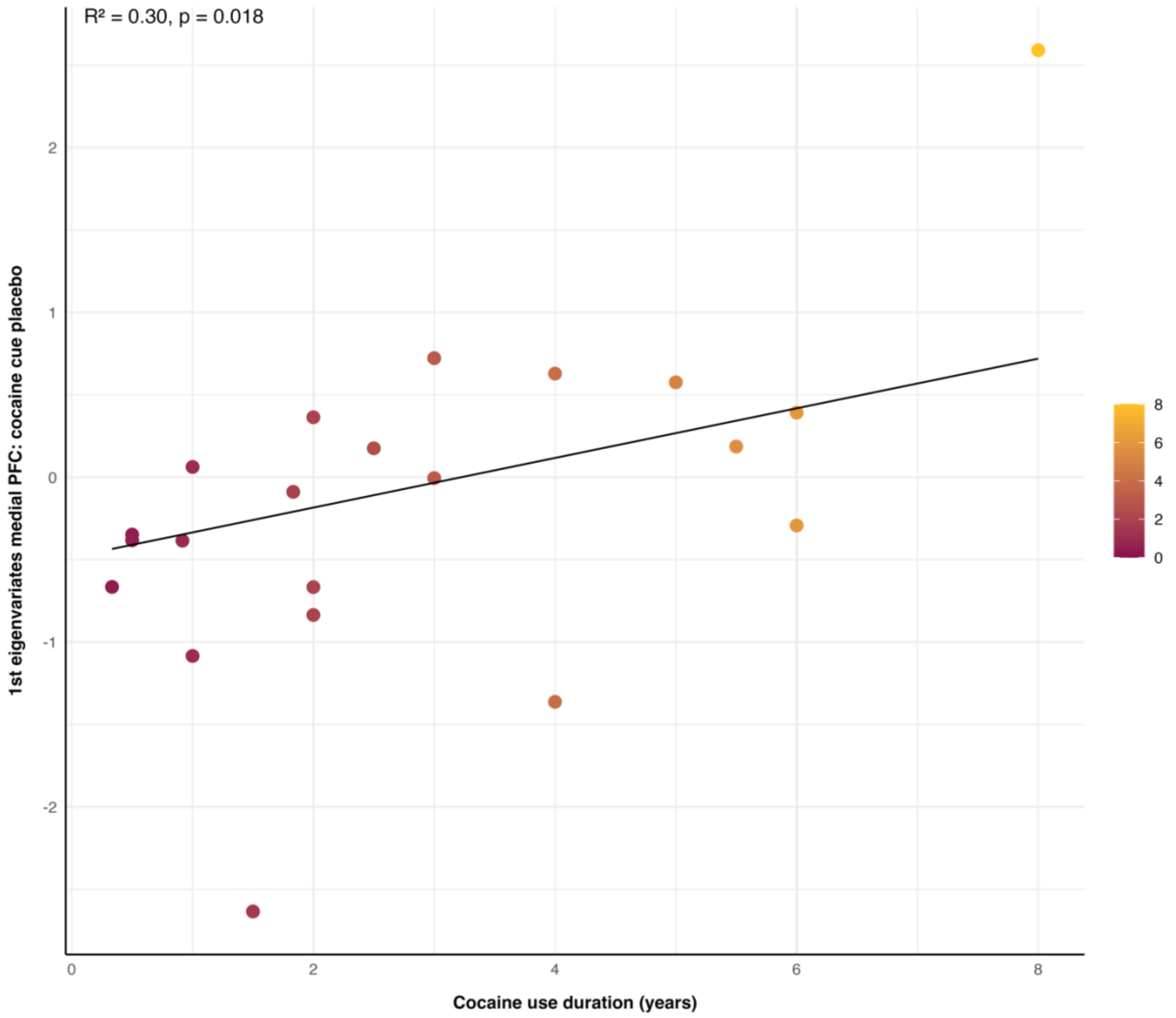

Figure S2. Prediction of cocaine cue reactivity in the medial prefrontal cortex (PFC) by the duration of cocaine use after exclusion of extreme value. Robust regression model based on LTS estimates, revealed a medium prediction of BOLD signal in the medial PFC when exposed to cocaine cues. The color shading on the graph represents the duration of cocaine use in years.

When testing the prediction of reactivity to cocaine cues compared to neutral cues in the placebo condition (cocaine cue placebo > neutral cue placebo) similar results to those observed for the BOLD response during cocaine cue exposure were found. This relationship was modelled using a robust regression model based on Huber estimation, which offered the best fit for the data: eigenvariates SFG = -0.61 + 0.17 *(cocaine use duration), R2 = 0.19, t = 2.067, p = 0.026 one-tailed.

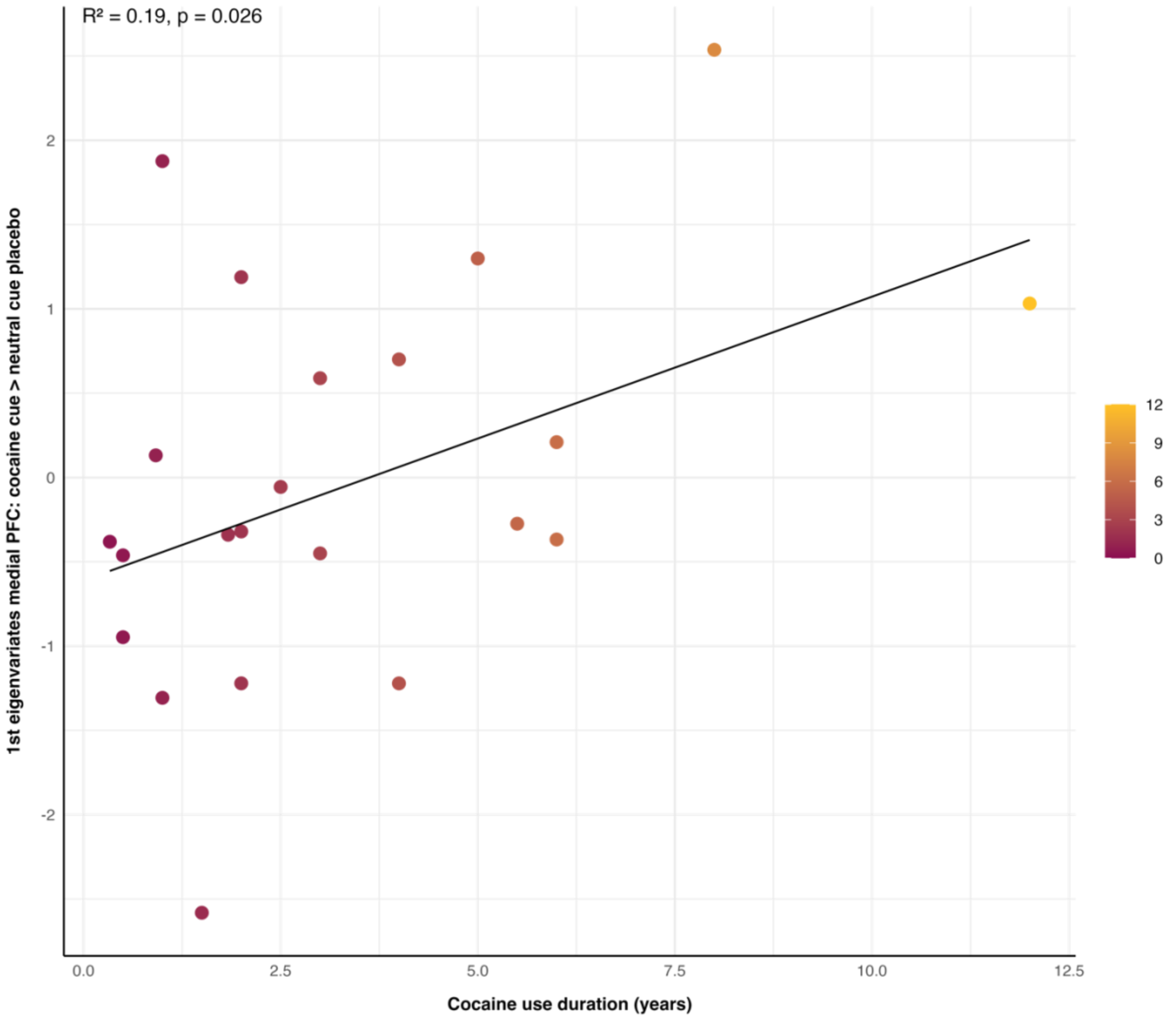

Figure S3. Prediction of cocaine cue reactivity in the medial prefrontal cortex (PFC) by the duration of cocaine use. Robust regression model based on Huber estimates, revealed a weak prediction of BOLD signal in the medial PFC when exposed to cocaine cues compared to neutral cues in the placebo condition. The color shading on the graph represents the duration of cocaine use in years.

References

1. Hulka LM, Scheidegger M, Vonmoos M, Preller KH, Baumgartner MR, Herdener M, Seifritz E, Henning A, Quednow BB. Glutamatergic and neurometabolic alterations in chronic cocaine users measured with (1) H-magnetic resonance spectroscopy. *Addiction Biology* (2014) 21:205–217. doi: 10.1111/adb.12217

2. Engeli EJE, Zoelch N, Hock A, Nordt C, Hulka LM, Kirschner M, Scheidegger M, Esposito F, Baumgartner MR, Henning A, et al. Impaired glutamate homeostasis in the nucleus accumbens in human cocaine addiction. *Mol Psychiatr* (2020)1–9. doi: 10.1038/s41380-020-0828-z
